# Supplementary material for: Ranking and selecting hospital information systems: A multi-criteria decision making approach using TOPSIS in Hamadan, Iran
Source: PLOS Digit Health. 2025 Sep 23;4(9):e0001016. doi: 10.1371/journal.pdig.0001016 (PMC12456787; doi:10.1371/journal.pdig.0001016)
Supplement: Attachment — Submitted filename: review.docx [file pdig.0001016.s001.docx]

**1. Overview and Main Claims**

The manuscript presents a study evaluating and ranking three Hospital Information Systems (HIS)—PARDAZESHGARAN, SAYAN, and RAYAVARAN—used in teaching hospitals affiliated with Hamadan University of Medical Sciences, Iran. The study employs the Technique for Order of Preference by Similarity to Ideal Solution (TOPSIS), a multi-criteria decision-making (MCDM) method, to rank the systems based on eight criteria: technical quality, software quality, support quality, workflow support quality, output quality, cost, user satisfaction, and interdepartmental communication quality. The main claims are:

- Cost and output quality are the most critical criteria for HIS evaluation.
- PARDAZESHGARAN ranks highest, followed by SAYAN and RAYAVARAN.
- The TOPSIS method provides a systematic and objective framework for HIS evaluation.

The study is relevant to the field of digital health, as HIS are integral to modern healthcare systems, and systematic evaluation methods can guide decision-making for system selection and improvement.

**2. Evaluation Against PLOS Digital Health Criteria**

**Originality**

The study applies TOPSIS, a well-established MCDM method, to evaluate HIS in a specific Iranian context, which adds some novelty to the application of operations research in healthcare informatics. However, the use of TOPSIS for HIS evaluation is not entirely novel, as referenced studies (e.g., Huang, 2008; Ma et al., 2022) have used TOPSIS in similar contexts. The manuscript acknowledges prior work but could better articulate how its specific focus on Hamadan’s teaching hospitals and the selected criteria contribute to the literature. For instance, the authors could highlight whether the criteria weighting or the local context introduces unique insights compared to previous studies.

**Recommendation**: Strengthen the originality claim by explicitly discussing how this study’s context, criteria selection, or findings differ from or complement existing TOPSIS-based HIS evaluations. If applicable, cite any unique challenges or characteristics of the Iranian healthcare system that make this study distinct.

**Importance and Broad Interest**

The topic is highly relevant to researchers, clinicians, and healthcare administrators in digital health, particularly in resource-constrained settings where HIS selection impacts cost, efficiency, and care quality. The emphasis on cost and output quality aligns with global priorities for optimizing healthcare technology investments. However, the study’s focus on three hospitals in Hamadan limits its immediate applicability to broader contexts. The findings could interest those working on HIS evaluation in similar settings, but the manuscript would benefit from discussing how the methodology or insights could be generalized or adapted elsewhere.

**Recommendation**: Expand the discussion to address how the methodology or findings could apply to other regions or healthcare systems. Highlight the broader implications for digital health stakeholders, such as policymakers or HIS vendors.

**Methodological Rigor and Ethical Standards**

The study employs a cross-sectional design with a clear methodology:

- **Criteria Selection**: Eight criteria were identified through literature review and validated by an expert panel, with a threshold of 3.5/5 for acceptance. The process is transparent, though the rationale for selecting these specific criteria over others (e.g., security, scalability) could be clearer.
- **Expert Panels**: Two panels (14 for criteria weighting, 6 for HIS evaluation) were carefully selected based on experience and expertise. The demographic data and selection criteria are well-documented, enhancing reproducibility.
- **TOPSIS Application**: The TOPSIS method is appropriately applied, with detailed steps (normalization, weighted decision matrix, ideal solutions, and relative closeness) and supporting tables. Excel was used for calculations, which is acceptable for this scale but limits scalability for larger datasets.
- **Ethical Considerations**: The study was approved by an ethics committee (IR.UMSHA.REC.1399.697), and informed consent was obtained, meeting ethical standards.

**Limitations**:

- The sample size (14 and 6 experts) is small, which the authors acknowledge as a limitation. This raises concerns about the robustness of criteria weights and system evaluations.
- The cross-sectional design limits insights into long-term HIS performance.
- The questionnaire’s development is described, but its full content or validation process (e.g., pilot testing, reliability analysis) is not detailed, which could affect the rigor of data collection.

**Recommendations**:

1. Provide more detail on the questionnaire’s development, including any validation steps or reliability measures (e.g., Cronbach’s alpha).
2. Justify the choice of the eight criteria and explain why other potentially relevant criteria (e.g., data security, interoperability) were excluded.
3. Consider sensitivity analysis to test the robustness of TOPSIS rankings against variations in criteria weights, addressing the small sample size concern.

**Substantial Evidence for Conclusions**

The study provides sufficient evidence for its conclusions:

- **Criteria Weights**: Table 1 and Table 2 show that cost and output quality received the highest weights (0.133 each), supported by expert scores. The process for calculating weights is transparent.
- **HIS Rankings**: The TOPSIS analysis (Tables 3–7) clearly demonstrates PARDAZESHGARAN’s superior performance (Cl = 0.959), followed by SAYAN (0.516) and RAYAVARAN (0.474). The decision matrix and weighted calculations are well-documented.
- **Alignment with Literature**: The discussion compares findings with prior studies, reinforcing the importance of cost, output quality, and support quality.

However, the small expert sample size and limited hospital scope weaken the generalizability of the rankings. Additionally, the manuscript notes discrepancies with Meidani et al. (2013), where RAYAVARAN ranked higher, but does not fully explore potential reasons beyond methodological differences (e.g., system updates, hospital-specific factors).

**Recommendation**: Discuss potential reasons for discrepancies with prior studies, such as changes in HIS versions or contextual factors. Acknowledge the limitations of the small sample size more explicitly in the conclusion and suggest how future studies could validate the findings.

**Utility and Accessibility**

The study’s utility lies in its systematic approach to HIS evaluation, which can guide hospital administrators in selecting systems. The TOPSIS method is accessible to those familiar with MCDM, and the use of Excel makes it feasible for resource-limited settings. However, the manuscript does not discuss the practical implementation of the findings (e.g., how hospitals can adopt PARDAZESHGARAN or improve SAYAN/RAYAVARAN). Additionally, the criteria weights and rankings are context-specific, and the manuscript could better address how other institutions could adapt the methodology.

**Recommendation**: Include a section or paragraph on practical applications, such as how hospitals can use the TOPSIS framework or prioritize criteria like cost and output quality. Provide guidance on adapting the methodology for different contexts.

**Open Science Standards**

- **Data Availability**: The manuscript states that data are available upon the journal’s request, which partially meets PLOS’s policy of full data availability. However, the data are not deposited in a public repository, which limits transparency.
- **Software Availability**: The study used Excel for TOPSIS calculations, which is widely available, but no custom scripts or templates are shared.
- **Reporting Guidelines**: The study does not explicitly mention adherence to reporting guidelines (e.g., STROBE for cross-sectional studies), though it follows a structured format.
- **Reproducibility**: The methodology is detailed enough to allow reproduction, but sharing the questionnaire and raw data would enhance this.

**Recommendations**:

1. Deposit the anonymized dataset (e.g., expert scores, decision matrices) in a public repository (e.g., Figshare) and provide an accession number.
2. Share the Excel template or TOPSIS calculation steps as supplementary material to aid replication.
3. Confirm adherence to relevant guidelines (e.g., STROBE) or explain why they were not applicable.

**3. Additional Considerations**

**Literature Context and Fairness**

The manuscript cites relevant studies (e.g., Farzandipour et al., 2017; Ker et al., 2018) to contextualize its findings and criteria. The literature review is comprehensive, covering HIS evaluation, TOPSIS applications, and related challenges. However, the discussion could better integrate these references to highlight how this study builds on or diverges from prior work. The treatment of the literature appears fair, with no evident bias.

**Recommendation**: Strengthen the discussion by synthesizing how this study’s findings align with or challenge specific prior studies, particularly regarding criteria prioritization and TOPSIS outcomes.

**Clarity and Organization**

The manuscript is well-organized, with clear sections (Introduction, Materials and Methods, Results, Discussion, Conclusion). The writing is generally clear, though some sections (e.g., TOPSIS steps) could be more concise. Tables are well-presented and support the narrative. The abstract and author summary effectively summarize the study, though the abstract could mention the small sample size limitation.

**Recommendations**:

1. Streamline the TOPSIS methodology section by referring readers to cited references for standard steps, focusing on study-specific adaptations.
2. In the abstract, briefly note the sample size limitation to set realistic expectations.

**Potential for Revision**

The study shows strong potential but would benefit from revisions to address the small sample size, data availability, and generalizability. The core methodology and findings are sound, and with enhancements, the manuscript could be suitable for publication.

**Recommendation**: Encourage the authors to revise and resubmit, focusing on the recommendations below.

**4. Specific Comments for Authors**

**Strengths**

- The use of TOPSIS provides a robust, systematic approach to HIS evaluation, which is valuable for decision-makers.
- The study is well-structured, with clear documentation of methods, results, and tables.
- The focus on cost and output quality aligns with practical priorities in healthcare settings.
- Ethical approval and expert panel selection enhance credibility.

**Major Revisions**

1. **Sample Size and Generalizability**: Acknowledge the small expert sample size (14 and 6) more prominently in the abstract and conclusion. Conduct a sensitivity analysis to test the robustness of TOPSIS rankings or discuss how a larger sample could validate the findings.
2. **Data Availability**: Deposit the anonymized dataset in a public repository and provide an accession number to meet PLOS’s open science policy. Share the Excel template or TOPSIS steps as supplementary material.
3. **Criteria Justification**: Clarify why the eight criteria were chosen and why others (e.g., security, interoperability) were excluded. Detail the questionnaire’s validation process.
4. **Practical Implications**: Add a section on how hospitals can apply the findings (e.g., adopting PARDAZESHGARAN, improving SAYAN/RAYAVARAN) and adapt the methodology for other contexts.
5. **Literature Integration**: Strengthen the discussion by comparing findings with specific prior studies and explaining discrepancies (e.g., Meidani et al., 2013).

**Minor Revisions**

1. **Abstract**: Mention the sample size limitation briefly.
2. **TOPSIS Section**: Condense standard TOPSIS steps and focus on study-specific details.
3. **Reporting Guidelines**: Confirm adherence to STROBE or explain its inapplicability.
4. **Clarity**: Proofread for minor grammatical errors (e.g., “Data Avallability” on Page 4) and ensure consistency in terminology (e.g., “Communication Quality Between Departments” vs. “interdepartmental communication quality”).
