# Supplementary material for: Ranking and selecting hospital information systems: A multi-criteria decision making approach using TOPSIS in Hamadan, Iran
Source: PLOS Digit Health. 2025 Sep 23;4(9):e0001016. doi: 10.1371/journal.pdig.0001016 (PMC12456787; doi:10.1371/journal.pdig.0001016)
Supplement: Attachment — Submitted filename: PDIG-D-25-00100.docx [file pdig.0001016.s002.docx]

**Review of "Ranking and Selecting Hospital Information Systems: A Multi-Criteria Approach Using TOPSIS in Hamadan, Iran"**

**Overall Assessment**

The manuscript is a Multi-Criteria Approach Using TOPSIS in Hamadan, Iran" (PDIG-D-25-00100) submitted to *PLOS Digital Health*. As a reviewer, I find the manuscript to be a valuable contribution to the field of health informatics, addressing a critical need for systematic evaluation of Hospital Information Systems (HIS) in the context of Iranian teaching hospitals. The use of the TOPSIS method to rank HIS based on multi-criteria decision-making is novel and relevant, aligning well with the journal’s focus on advancing digital health technologies. However, the manuscript has several significant issues that require substantial revisions to meet the standards of *PLOS Digital Health*. Based on the importance of the topic and the potential for improvement, I recommend **acceptance with major revisions**. Below, I outline the key strengths, issues, and specific revisions needed to ensure the manuscript’s suitability for publication.

The manuscript addresses a timely and relevant topic by evaluating HIS, a cornerstone of digital health, using a rigorous multi-criteria decision-making approach (TOPSIS). Its focus on Iranian teaching hospitals fills a gap in the literature, where systematic HIS evaluation studies are limited. The study’s methodology, including the use of expert panels and literature-based criteria (e.g., cost, output quality, support quality), is well-grounded and demonstrates scientific rigor. The clear structure, academic tone, and alignment with ethical standards (e.g., ethical approval, informed consent) further support its potential for publication. The findings, particularly the ranking of PARDAZESHGARAN as the top HIS and the emphasis on cost and output quality, provide actionable insights for healthcare administrators.

**Major Issues**

Despite its strengths, the manuscript has several critical issues that must be addressed to ensure scientific accuracy, transparency, and impact. These issues necessitate major revisions, as they involve methodological corrections, enhanced clarity, and deeper interpretation.

**Methodological Errors in TOPSIS Analysis**:

- A critical error exists in Table 5 (p. 15, p. 33), where the positive and negative ideal solutions for the cost criterion are reversed. As cost is a negative criterion (lower is better), the positive ideal should be the lowest value (0.075), and the negative ideal should be the highest (0.079). This error likely affects the distances (Table 6) and relative closeness (Table 7), potentially altering the final rankings. This undermines the study’s validity and requires recalculation.
- The TOPSIS methodology description in the methods section is incomplete, covering only normalization and weighted matrix steps. Key steps, such as calculating distances to ideal solutions and relative closeness (CI), are omitted, reducing reproducibility.

**Limited Methodological Transparency**:

- The questionnaire development process lacks detail on the literature review or frameworks used (e.g., DeLone and McLean model, ISO standards), limiting transparency.
- The small sample sizes (14 experts for criteria weighting, 6 for HIS evaluation, and three hospitals) are acknowledged as a limitation but not justified. A rationale citing practical constraints or similar studies is needed.
- The ethical considerations section is brief, lacking specifics on consent procedures (e.g., written or verbal) and data protection measures (e.g., anonymization protocols).

**Clarity and Presentation Issues**:

- The results section lacks a narrative explanation of the TOPSIS process, making it difficult for readers unfamiliar with MCDM to follow how scores lead to rankings.
- An incorrect reference to Table 9 (p. 17, line 287) instead of Table 7 in the discussion risks confusing readers and indicates a need for careful proofreading.
- The absence of figures (e.g., a bar chart or radar plot comparing HIS scores) limits the visual communication of results, which is a missed opportunity for a journal emphasizing clear data presentation.

**Weaknesses in Discussion and Conclusion**:

- The discussion overinterprets small differences in criteria weights (e.g., technical quality: 0.112 vs. user satisfaction: 0.121) as evidence that users prioritize practical outcomes, requiring a more nuanced interpretation.
- The discussion does not sufficiently explore discrepancies with prior studies or discuss practical implications for stakeholders, limiting the study’s impact.
- The conclusion is brief and does not articulate the broader applicability of the findings or include a call to action for stakeholders.

**Minor Issues**

**Inconsistent Terminology**:

- The manuscript uses “multi-criteria approach” in the title and abstract but occasionally refers to “multi-criteria decision-making” elsewhere (e.g., introduction, p. 7). Consistent use of “multi-criteria decision-making” (MCDM), the standard term in operations research, would improve professionalism.
- The HIS name “Rayavaran” is inconsistently capitalized (e.g., “RAYAVARAN” in Table 3, p. 32, vs. “Rayavaran” in the text, p. 16). This should be standardized throughout.

**Formatting Inconsistencies in Tables**:

- The decimal places in tables are inconsistent. For example, Table 2 (p. 31) reports weights to three decimal places (e.g., 0.133), while Table 5 (p. 33) uses three or four decimal places (e.g., 0.075 vs. 0.0792). All numerical values should use a consistent number of decimal places (e.g., three decimals throughout).
- Table titles and captions could be more descriptive. For instance, Table 1 (p. 31) is titled “Average Scores for Key HIS Criteria,” but it could specify “Based on Expert Panel Evaluation” to clarify the source.

**Minor Proofreading Errors**:

- There are minor grammatical errors, such as “hospitals lacking robust HIS may struggle to remain competitive” (p. 7, introduction), which could be rephrased for clarity (e.g., “Hospitals without robust HIS may face challenges in maintaining competitiveness”).
- The phrase “data are ready for delivery following the journal’s request” (p. 4) is awkward. A clearer phrasing would be “Data are available upon journal request” or “Data will be provided as per journal requirements.”

**Lack of Footnotes in Tables**:

- Tables 1–7 lack footnotes explaining key terms or methods, such as the 5-point Likert scale (Table 1), normalization process (Table 4), or ideal solutions (Table 5). Adding brief footnotes would enhance accessibility for readers unfamiliar with TOPSIS or the study’s methods.

**Vague Wording in Abstract**:

- The abstract’s mention of “sample size” as a limitation (p. 1) is vague. Specifying whether this refers to the number of hospitals, experts, or systems evaluated would improve clarity without requiring major revision.
- The term “expert consultation” in the abstract is imprecise. A minor clarification, such as “expert panel scoring,” would align with the methods section.

**Underdeveloped Contextual Details**:

- The introduction briefly mentions the Iranian healthcare context but lacks specific details, such as HIS adoption rates or challenges in teaching hospitals. Adding a sentence or two with relevant statistics or context would strengthen the rationale without requiring major restructuring.
- The demographic data of the expert panels (p. 12, p. 30) are presented but not integrated into the narrative. A minor addition explaining their relevance (e.g., “The panel’s expertise ensured robust criteria weighting”) would enhance clarity.

**Limited Use of Abbreviations**:

- The manuscript introduces “Hospital Information Systems (HIS)” but does not consistently use the abbreviation throughout (e.g., “hospital information systems” appears in full in the discussion, p. 17). Consistently using “HIS” after its first introduction would improve readability.

**Minor Redundancy in Discussion**:

- The discussion repeats the importance of cost and output quality (p. 17) without adding new insights in some sentences. Streamlining these points (e.g., combining redundant statements) would improve conciseness.
