# Supplementary material for: Ranking and selecting hospital information systems: A multi-criteria decision making approach using TOPSIS in Hamadan, Iran
Source: PLOS Digit Health. 2025 Sep 23;4(9):e0001016. doi: 10.1371/journal.pdig.0001016 (PMC12456787; doi:10.1371/journal.pdig.0001016)
Supplement: Attachment — Submitted filename: Rebuttal Letter.docx [file pdig.0001016.s004.docx]

**Rebuttal Letter**

**Subject: Response to Reviewer Comments – PDIG-D-25-00100- "Ranking and Selecting Hospital Information Systems: A Multi-Criteria Approach Using TOPSIS in Hamadan, Iran"**

Dear Editor Plos Digital Health

Thank you for the opportunity to revise our manuscript, "Ranking and Selecting Hospital Information Systems: A Multi-Criteria Approach Using TOPSIS in Hamadan, Iran," and for the insightful feedback provided by the reviewer. We appreciate the thorough evaluation and believe that addressing these comments has significantly strengthened our manuscript.

We have carefully considered all suggestions and made revisions accordingly. Our responses to each point are detailed below. We have also highlighted all changes in the revised manuscript for easier identification.

**Reviewer Comment: Major Revisions - Sample Size and Generalizability - "Acknowledge the small expert sample size (14 and 6) more prominently in the abstract and conclusion. Conduct a sensitivity analysis to test the robustness of TOPSIS rankings or discuss how a larger sample could validate the findings."**

**Response:** We appreciate this important feedback regarding the sample size and generalizability. We have taken the following actions:

- **Prominent Acknowledgment:** The small expert sample size and the study's localized context are now explicitly acknowledged in the Abstract and more prominently in the Conclusion, setting realistic expectations for the generalizability of our findings.
- **Sensitivity Analysis Discussion:** While a full sensitivity analysis was beyond the scope of this initial study, we have added a dedicated "Sensitivity Analysis Considerations" subsection within the Methods. Here, we transparently discuss the implications of the small expert sample size and suggest that future research should explore sensitivity analysis (e.g., varying weights or using bootstrap resampling) to assess rank stability. This acknowledges the limitation and provides clear direction for future work.
- **Location of Changes:**
  - **Abstract:** Revised to include the sample size limitation.
  - **Conclusion:** Paragraph 2.
  - **Materials and Methods - "Sensitivity Analysis Considerations" subsection:** New subsection added.

**Reviewer Comment: Major Revisions - Data Availability - "Deposit the anonymized dataset in a public repository and provide an accession number to meet PLOS’s open science policy. Share the Excel template or TOPSIS steps as supplementary material."**

**Response:** We fully agree with the importance of Open Science practices and data transparency.

- **Data Availability:** We deposited the anonymized dataset (expert scores, decision matrices) in a publicly accessible repository, such as Figshare. We then update the Data Availability Statement in the submission system to include the accession number.
  - The anonymized dataset generated and analyzed during the current study is available in the: Mohammadpour, Ali (2025). HIS Ranking data and topsis.xlsx. figshare. https://figshare.com/articles/dataset/_/29260397
- **Software/Methodology Availability:** We will also prepare and share the Excel file containing the TOPSIS calculation steps as supplementary material to aid replication and transparency.
- **Location of Changes:**
  - **Data Availability Statement:** This be updated in the manuscript submission system.
  - **Supplementary Materials:** To be added.

**Reviewer Comment: Major Revisions - Criteria Justification - "Clarify why the eight criteria were chosen and why others (e.g., security, interoperability) were excluded. Detail the questionnaire’s validation process."**

**Response:** We have enhanced the "Data Collection and Criteria Selection" subsection in the Methods to provide more clarity on these points.

- **Criteria Justification:** We now justify the choice of the eight criteria by emphasizing their recurring importance in the literature and their practical relevance to the specific operational and strategic priorities of HIS in Iranian university hospitals. We also briefly acknowledge that while other criteria (like data security and interoperability) are vital, our study's focus was narrowed to those most frequently highlighted by our experts as critical for overall system performance and direct user experience within this particular context.
- **Questionnaire Validation:** We have added more explicit details regarding the questionnaire's development and validation, specifying that content validity was ensured through expert review. We also **performed a Cronbach's alpha (α=0.905).**
- **Location of Changes:**
  - **Materials and Methods - "Data Collection and Criteria Selection" subsection:** See new bullet points "Justification for Criteria Selection" and "Questionnaire Validation".

**Reviewer Comment: Major Revisions - Practical Implications - "Add a section on how hospitals can apply the findings (e.g., adopting PARDAZESHGARAN, improving SAYAN/RAYAVARAN) and adapt the methodology for other contexts."**

**Response:** We agree that practical implications are crucial for the utility of the study. We have added a dedicated "Practical Implications for Hospitals and Policymakers" subsection within the Discussion. This section explicitly outlines how hospitals can utilize the TOPSIS framework, prioritize criteria based on our findings, and apply the insights for informed decision-making. We also detail how the methodology can be adapted for different contexts and how it can benefit various digital health stakeholders.

- **Location of Changes:**
  - **Discussion - "Utility of TOPSIS and Practical Implications" subsection:** Added a new sub-heading "Practical Implications for Hospitals and Policymakers" with detailed bullet points.
  - **Conclusion:** Paragraph 2.

**Reviewer Comment: Major Revisions - Literature Integration - "Strengthen the discussion by comparing findings with specific prior studies and explaining discrepancies (e.g., Meidani et al., 2013)."**

**Response:** We have significantly strengthened the discussion regarding the integration of our findings with existing literature.

- **Synthesis with Prior Studies:** Discussion section: Paragraphs 1, 2, and 3 now provides a more robust synthesis of how our identified criteria (cost, output quality, support, workflow support) converge with previous research, reinforcing their universal importance.
- **Explanation of Discrepancies:** In Discussion Section Paragraphs 4, we have significantly expanded the discussion on discrepancies with prior studies, particularly concerning the Meidani et al. (2013/2015) study. We now offer several potential reasons beyond mere methodological differences, including: changes in HIS versions, contextual factors and hospital-specific needs, and differences in evaluation criteria and weighting. This provides a more comprehensive explanation for observed divergences.
- **Location of Changes:**
  - **Discussion -** Discussion section: Paragraphs 1, 2, and 3
  - **Discussion -** Discussion section: Paragraphs 4.

**Reviewer Comment: Minor Revisions - Abstract - "Mention the sample size limitation briefly."**

**Response:** As noted in our response to the "Major Revisions" comments, the Abstract has been revised to include a brief, explicit mention of the sample size limitation, setting clear expectations for readers from the outset.

- **Location of Changes:**
  - **Abstract:** Revised to include the sample size limitation.

**Reviewer Comment: Minor Revisions - TOPSIS Section - "Condense standard TOPSIS steps and focus on study-specific details."**

**Response:** We have reviewed the "TOPSIS Analysis" subsection in the Methods to ensure it is concise while maintaining sufficient clarity on the study-specific adaptations of the TOPSIS steps. We believe the current level of detail is appropriate for reproducibility without being overly verbose, balancing explanation with referring to standard methods.

- **Location of Changes:**
  - **Materials and Methods - "TOPSIS Analysis" subsection:** Reviewed for conciseness while maintaining clarity.

**Reviewer Comment: Minor Revisions - Reporting Guidelines - "Confirm adherence to STROBE or explain its inapplicability."**

**Response:** We confirm that our study adheres to the principles of good research reporting for cross-sectional studies. While we did not explicitly cite a specific guideline (like STROBE) in the text, our methodology and reporting align with standard practices for such studies. We have ensured a structured and transparent presentation of our methods and results to facilitate reproducibility.

- **Location of Changes:** Implicitly addressed by the structure and detail in Methods and Results sections.

**Reviewer Comment: Minor Revisions - Clarity - "Proofread for minor grammatical errors (e.g., “Data Avallability” on Page 4) and ensure consistency in terminology (e.g., “Communication Quality Between Departments” vs. “interdepartmental communication quality”)."**

**Response:** We have thoroughly proofread the entire manuscript to correct any minor grammatical errors and ensure consistency in terminology throughout. Specifically, we have corrected the typo "Data Avallability" and ensured consistent use of "interdepartmental communication quality" as requested.

- **Location of Changes:** Implemented throughout the revised manuscript, particularly in the Introduction, Methods, and Results sections where terminology was adjusted.

We sincerely thank the reviewer for their valuable time and constructive comments. We believe that incorporating these suggestions has significantly improved the clarity, rigor, and overall impact of our manuscript. We hope that the revised manuscript is now suitable for publication in PLOS Digital Health.

Sincerely,

Dr. Ali Mohammadpour

Corresponding Author
